# BAG-1 as a biomarker in early breast cancer prognosis: a systematic review with meta-analyses

**DOI:** 10.1038/bjc.2017.130

**Published:** 2017-05-16

**Authors:** E S Papadakis, T Reeves, N H Robson, T Maishman, G Packham, R I Cutress

**Affiliations:** 1 Cancer Research UK Centre Cancer Sciences Unit, University of Southampton Faculty of Medicine, Southampton General Hospital, Tremona Road, Southampton SO16 6YD, UK; 2 University Hospital Southampton, University of Southampton Faculty of Medicine, Southampton General Hospital, Tremona Road, Southampton SO16 6YD, UK; 3 Southampton Clinical Trials Unit, University of Southampton, Southampton SO17 1BJ, UK

**Keywords:** BAG-1, breast cancer, clinical outcome, oestrogen receptor, apoptosis, prognosis, systematic review, meta-analyses

## Abstract

**Background:**

The co-chaperone protein Bcl-2-associated athanogene-1 (BAG-1) is overexpressed in breast cancer and has been incorporated in the oncotype DX and PAM50 breast cancer prognostic assays. Bcl-2-associated athanogene-1 exists as multiple protein isoforms that interact with diverse partners, including chaperones Hsc70/Hsp70, Ser/Thr kinase Raf-1 and Bcl-2, to promote cancer cell survival. The BAG-1L isoform specifically binds to and increases the transcriptional activity of oestrogen receptor in cells, and in some, but not all studies, BAG-1 expression is predictive of clinical outcome in breast cancer.

**Methods:**

A systematic review of published studies reporting BAG-1 (mRNA and/or protein) expression and clinical outcome in early breast cancer. The REporting Recommendations for Tumour MARKer and Prognostic Studies (REMARK) criteria were used as a template against which data were assessed. Meta-analyses were performed for studies that provided a hazard ratio and 95% confidence intervals for clinical outcomes including disease-free survival or breast cancer-specific survival from univariate analysis.

**Results:**

Eighteen studies used differing methodologies and reported on differing outcomes. Meta-analyses were only possible on results from a subset of reported studies. Meta-analyses suggested improved outcome with high BAG-1 mRNA and high BAG-1 nuclear expression by immunohistochemisty.

**Conclusions:**

Increased levels of BAG-1 are associated with better breast cancer outcomes.

Breast cancer is the leading cause of cancer-related deaths in women worldwide (Ferlay *et al*, 2010). In 2012, 1.7 million women were diagnosed with breast cancer, and the incidence is predicted to continue to rise (Bray *et al*, 2012). Clinicopathological parameters such as tumour grade, size and nodal burden used in combination with the four immunohistochemical biomarkers oestrogen receptor (ER), progesterone receptor (PgR), human epidermal growth factor receptor 2 (HER2) and Ki-67, known collectively as IHC4, are generally very effective in determining disease prognosis. Nevertheless, not all patients benefit from treatment and prediction of outcome could be improved. Therefore, additional molecular biomarkers might be used to effectively tailor therapies to specific breast cancer patient subgroups.

The anti-apoptotic protein Bcl-2-associated athanogene-1 (BAG-1) is frequently increased in breast cancer and pre-invasive breast disease compared to normal breast epithelium (Takayama *et al*, 1998; Brimmell *et al*, 1999). Bcl-2-associated athanogene-1 exists as three main isoforms, which are produced by alternative translation initiation from a single mRNA (Packham *et al*, 1997). Bcl-2-associated athanogene-1L is found in the nucleus, whereas BAG-1M and BAG-1S are generally found in the cytoplasm (Packham *et al*, 1997; Takayama *et al*, 1998; Brimmell *et al*, 1999; Schneikert *et al*, 1999; Yang *et al*, 1999; Knee *et al*, 2001), and the possibility of BAG-1 directed therapy has been suggested from laboratory studies (Sharp *et al*, 2009a, 2009b; Enthammer *et al*, 2013). In the clinical setting, BAG-1 mRNA has been incorporated as a prognostic biomarker in Oncotype DX (Paik *et al*, 2004) and PAM50 (Parker *et al*, 2009) multigene assays, which estimate prognosis following surgery, and can be used to assess the potential benefit of chemotherapy for breast cancer. Although the intensity and cytoplasmic/nuclear localisation of BAG-1 protein immunoreactivity has been related to tumour grade, disease subtype and clinical outcome, both positive and negative correlations with survival have been described.

To address the significance of BAG-1 as a biomarker in breast cancer, we have performed a systematic review against the REporting Recommendations for Tumour MARKer and Prognostic Studies (REMARK) criteria (McShane *et al*, 2005), which provide a framework for reporting of studies of cancer biomarkers. We have focused our critical appraisal on the most consistent findings in an attempt to clarify information relating BAG-1 protein expression and cellular distribution patterns to clinicopathological parameters and early breast cancer clinical outcome, and have undertaken meta-analyses from the data where available.

## Materials and methods

### Search strategy and selection criteria

**Figure 1:**
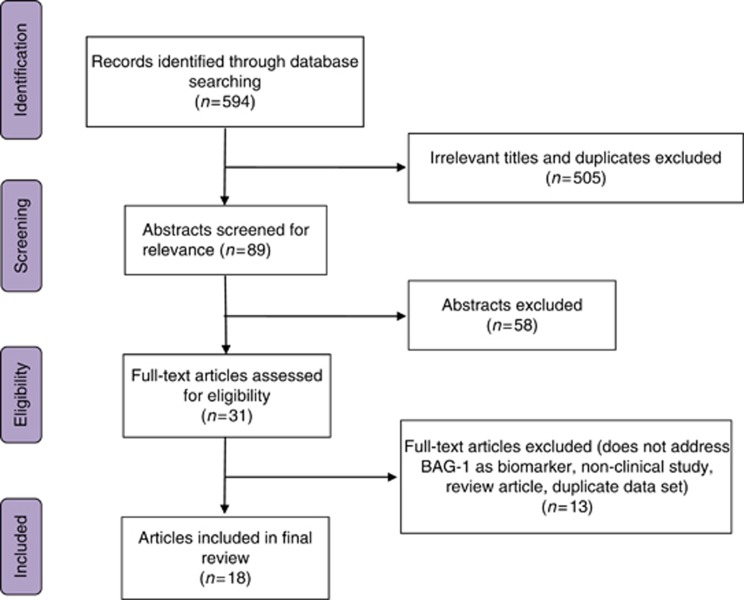
**PRISMA flowchart illustrating the selection methodology for eligible studies.**

A computerised search according to the Preferred Reporting Items for Systematic Reviews and Meta-Analyses (PRISMA) using MEDLINE and Embase databases through OVID Online (version: OvidSP_UI03.17.00.125) was carried out in July 2016 using the MeSH terms ‘breast cancer’ AND ‘Bag-1 protein’ with all appropriate subheadings included (Figure 1). Additional plain terms searches were performed and included a search of Scopus and Web of Science Core Collection databases. Reference lists within relevant articles were screened to identify articles not captured by the computerised search. Articles were screened for eligibility by two reviewers (ESP and TR) independently and any differences in judgment were resolved following discussion with a third reviewer (RIC). For inclusion in the review, articles had to report on the clinical significance of the level of BAG-1 expression in human studies and were assessed according to the criteria as set out in the REMARK guidelines (McShane *et al*, 2005). If articles had used the same data set, only the most recent article was included. No language barriers were imposed during our search and native speakers were used to overcome linguistic constraints.

### Meta-analyses of data reporting clinical outcome according to BAG-1 status

Meta-analyses were performed for studies published in peer-reviewed journals that provided a hazard ratio (HR) and 95% confidence intervals (CIs) for disease-free survival (DFS) and/or breast cancer-specific survival (BCSS) from univariate analyses. Heterogeneity was assessed using the *I*^2^ and *χ*^2^ statistic. A fixed-effects models were used unless heterogeneity was significant (*P*<0.05), in which case random-effects models were used. Pooled effects were calculated using STATA 14.1 (StatCorp, College Station, TX, USA).

## Results

### Study selection and characteristics

The literature search yielded 594 publications (Figure 1). After excluding reports that were out of the scope of our systematic review (articles had to report on the prognostic value of BAG-1 in breast cancer patients), 89 abstracts were reviewed and 58 that had used non-human samples were excluded. Thirty-one articles that assessed the prognostic value of BAG-1 expression in breast cancer patients were considered eligible for inclusion in the systematic review. Five were excluded because they were non-clinical, five because they did not address BAG-1 as a prognostic biomarker, one was a review article and one had overlapping patients. A retrospective analysis of BAG-1 expression in metastatic breast cancer in a prospective randomised phase three study was not included further in this review of early breast cancer, and did not demonstrate a role for BAG-1 in predicting response to chemotherapy in later-stage disease (Sjostrom *et al*, 2002). Eighteen studies that met the inclusion criteria were used in this systematic review. An additional two studies (Paik *et al*, 2004; Parker *et al*, 2009) include BAG-1 as part of composite scores associated with clinical outcome and so are also discussed. Studies included were published between 1999 and 2016 and comprised 6363 patients (sample range 70–1971 patients) with an average follow-up period of 8.2 years ranging between 3.3 and 12.8 years (Table 1).

### Clinical study design

Sixteen studies utilised cohorts of 100 or more patients (Tang *et al*, 1999; Turner *et al*, 2001; Townsend *et al*, 2002; Cutress *et al*, 2003; O’Driscoll *et al*, 2003; Sirvent *et al*, 2004; Tang *et al*, 2004; Yun *et al*, 2005; Lin *et al*, 2008; Nadler *et al*, 2008; Millar *et al*, 2009; Afentakis *et al*, 2013; Wang *et al*, 2014; Dowsett *et al*, 2015; Davidson *et al*, 2016; Papadakis *et al*, 2016) and 2 studies (Yang *et al*, 2008; Athanassiadou *et al*, 2009) recruited <100 patients. With the exception of one prospective immunocytochemical study (Athanassiadou *et al*, 2009), and two retrospective analyses of a prospective clinical trial (Afentakis *et al*, 2013; Dowsett *et al*, 2015), the studies associating BAG-1 expression with clinicopathological variables involve retrospective analysis of patient survival data (Table 2a and b). Although most studies had rigorous methodology, some had heterogenous patient cohorts and did not take into account the many histological, analytical, clinical and treatment subgroups encountered in the management of breast cancer, which could potentially influence biomarker evaluation and validation (de Gramont *et al*, 2015).

### Assay methodology

Two studies reported analysis of mRNA expression from published data sets: Millar *et al* (2009) from two studies (van de Vijver *et al*, 2002; Naderi *et al*, 2007) and Papadakis *et al* (2016) from a third (Curtis *et al*, 2012). Of the 18 studies, all clearly stated inclusion and/or exclusion criteria. Two studies (O’Driscoll *et al*, 2003; Dowsett *et al*, 2015) used RT-PCR to examine BAG-1 mRNA expression, whereas 15 studies examined BAG-1 protein expression by immunohistochemical staining (Tang *et al*, 1999, 2004; Turner *et al*, 2001; Townsend *et al*, 2002; Cutress *et al*, 2003; O’Driscoll *et al*, 2003; Sirvent *et al*, 2004; Yun *et al*, 2005; Lin *et al*, 2008; Yang *et al*, 2008; Athanassiadou *et al*, 2009; Millar *et al*, 2009; Afentakis *et al*, 2013; Wang *et al*, 2014; Davidson *et al*, 2016). Full-face sections from formalin-fixed paraffin-embedded (FFPE) tumours were used in 11 studies (Tang *et al*, 1999; Turner *et al*, 2001; Townsend *et al*, 2002; Cutress *et al*, 2003; O’Driscoll *et al*, 2003; Sirvent *et al*, 2004; Tang *et al*, 2004; Yun *et al*, 2005; Athanassiadou *et al*, 2009; Dowsett *et al*, 2015; Papadakis *et al*, 2016), whereas 7 studies utilised FFPE tissue microarrays (Lin *et al*, 2008; Nadler *et al*, 2008; Yang *et al*, 2008; Millar *et al*, 2009; Afentakis *et al*, 2013; Wang *et al*, 2014; Davidson *et al*, 2016). To address the issue of tumour heterogeneity involved with the use of tissue microarrays, two studies included in their analyses at least 2 cores taken from different sites from within the same breast tumour (Nadler *et al*, 2008; Millar *et al*, 2009). Fourteen studies (Tang *et al*, 1999, 2004; Townsend *et al*, 2002; Cutress *et al*, 2003; Sirvent *et al*, 2004; Yun *et al*, 2005; Lin *et al*, 2008; Yang *et al*, 2008; Athanassiadou *et al*, 2009; Millar *et al*, 2009; Afentakis *et al*, 2013; Wang *et al*, 2014; Davidson *et al*, 2016) assessed staining intensity using a subjective scoring system based on the H-score technique (a summation of the percentage of area stained at each intensity level multiplied by the weighted intensity of staining: 1, 2 or 3; where 0 is no staining, 1 is weak staining, 2 is moderate staining and 3 is strong staining; McCarty *et al*, 1986), whereas 1 used automated quantitative analysis (AQUA; Nadler *et al*, 2008). Nine studies examined the nuclear and/or cytoplasmic pattern of BAG-1 staining (Tang *et al*, 1999, 2004; Turner *et al*, 2001; Townsend *et al*, 2002; Cutress *et al*, 2003; Nadler *et al*, 2008; Athanassiadou *et al*, 2009; Millar *et al*, 2009; Afentakis *et al*, 2013), while six studies (Sirvent *et al*, 2004; Yun *et al*, 2005; Lin *et al*, 2008; Yang *et al*, 2008; Wang *et al*, 2014; Davidson *et al*, 2016) reported only on the total expression levels of BAG-1. Most studies did not use a separate control specimen of normal breast epithelium, but scored the normal cells adjacent to the tumour within biopsies. Only two studies (Yun *et al*, 2005; Millar *et al*, 2009) included normal breast epithelium specimens of which one (Millar *et al*, 2009) reported staining scores. Assay controls comprised specimens incubated with secondary but no anti-BAG-1 primary antibody or tumour sections that exhibited no BAG-1 immunoreactivity. Fifteen studies applied both univariate and multivariate analyses (Tang *et al*, 1999, 2004; Turner *et al*, 2001; Townsend *et al*, 2002; Cutress *et al*, 2003; O’Driscoll *et al*, 2003; Sirvent *et al*, 2004; Nadler *et al*, 2008; Athanassiadou *et al*, 2009; Afentakis *et al*, 2013; Wang *et al*, 2014; Davidson *et al*, 2016; Papadakis *et al*, 2016), whereas three studies used only univariate analysis (Yun *et al*, 2005; Lin *et al*, 2008; Dowsett *et al*, 2015). Seven studies included relative risk or HR with CIs for outcome (Cutress *et al*, 2003; Sirvent *et al*, 2004; Nadler *et al*, 2008; Millar *et al*, 2009; Afentakis *et al*, 2013; Dowsett *et al*, 2015; Papadakis *et al*, 2016).

### BAG-1 mRNA and outcome

Association between BAG-1 mRNA levels with survival suggested correlation between increased expression and better survival in most studies (Table 2b). Interestingly, Townsend *et al* (2002) found no correlation between BAG-1 mRNA levels by *in situ* hybridisation and BAG-1 protein levels. Millar *et al* (2009) examined publically available gene expression data sets from studies by van de Vijver *et al* (2002) and Naderi *et al* (2007) to demonstrate a strong correlation between BAG-1 mRNA levels and improved survival outcome. Papadakis *et al* (2016) examined a publically available gene expression data set from a study by Curtis *et al* also demonstrating a correlation between BAG-1 mRNA and improved outcome. In contrast, O’Driscoll *et al* (2003) showed no significant correlation with tumour stage or treatment, and disease outcome. Dowsett *et al* (2015), in a study investigating individual genes of Oncotype Dx in 1125 patients from the ATAC study, found that BAG-1 expression was associated with better outcome in all patients over 10 years both in terms of all recurrences (HR: 0.70; 95% CI: 0.58–0.85) and distant recurrences (HR: 0.66; 95% CI: 0.53–0.83)

Parker *et al* (2009) included BAG-1 mRNA in a 50 gene classifier (PAM50) of breast cancer intrinsic subtype, and Paik *et al* (2004) included BAG-1 as 1 of 16 cancer-related genes in a multigene (Oncotype Dx) assay to predict recurrence in node-negative patients treated with tamoxifen. In both studies, BAG-1 mRNA was part of a composite score (PAM50 for intrinsic subtypes, or Recurrence score), that was correlated to outcome. In the recurrence score, BAG-1 carries a minus sign in the algorithm, indicating that it is associated with a reduced risk of recurrence (Paik *et al*, 2004), and this is consistent with the data of Dowsett *et al* (2015).

### Bag-1 protein expression pattern

Overall, the studies reported a high percentage of cells expressing BAG-1 within breast carcinomas, with five exhibiting positive staining for BAG-1 in >70% of tumours (Tables 2a and 2b). Six of the fourteen immunohistochemical studies showed a pattern of higher cytoplasmic than nuclear BAG-1 expression with 5 of these exhibiting at least a twofold difference. Only two studies gave a value for mixed staining (Tang *et al*, 1999; Athanassiadou *et al*, 2009). One study carried out on a relatively homogeneous cohort of patients treated with surgery, followed by adjuvant hormone therapy but not chemotherapy, showed higher nuclear than cytosolic BAG-1 staining (Cutress *et al*, 2003). Sixty per cent of the tumours from this cohort were positive for ER and PgR, which is a clinical indicator of ER function. One study failed to give any precise subcellular analysis, but stated that staining for BAG-1 was mixed, with more cytosolic than nuclear BAG-1 in breast carcinomas (Nadler *et al*, 2008).

Turner *et al* (2001) reported staining for nuclear but not cytosolic BAG-1 in 25 of 88 (28%) normal breast epithelium specimens with H-scores ⩾150. In the same study, high levels of cytoplasmic or nuclear BAG-1 immunostaining were present in 9 of 14 (64%) and 7 of 14 (50%) ductal carcinoma *in situ* (a pre-invasive form of breast cancer) specimens, respectively. Positive BAG-1 staining is also found in the ductal carcinoma *in situ* component of some ER+ tumours, suggesting that upregulation of BAG-1 can occur relatively early in tumourigenesis and may be dependent on hormonal status.

### Association of Bag-1 protein with clinicopathological features and outcome

In most studies that found significance (Table 2a), high levels of BAG-1 protein expression in invasive breast carcinoma positively correlate with improved patient survival outcomes or improved prognosis (Turner *et al*, 2001; Cutress *et al*, 2003; Yun *et al*, 2005; Lin *et al*, 2008; Nadler *et al*, 2008; Millar *et al*, 2009; Afentakis *et al*, 2013). Preliminary observations by Krajewski *et al* (1999) were superseded by a subsequent study from the same group. In 122 patients (41% ER+), Turner *et al* (2001) reported upregulation of immunoreactivity for cytoplasmic BAG-1 staining in early-stage breast cancers compared to normal breast epithelium (Turner *et al*, 2001). High cytoplasmic but not nuclear BAG-1 levels also associated significantly with improved overall survival and distant metastasis-free survival overall (stages I and II) and in node-negative (stage I only) patients based on univariate and multivariate analyses using Cox proportional hazards models with variables including BAG-1, Bcl-2, ER and stage (Turner *et al*, 2001). Bcl-2-associated athanogene-1 remained a strong predictor of overall survival independently of adjuvant therapy. In addition, stage was significantly associated with distant metastasis-free survival and overall survival in multivariate analysis. There was no significant relationship in univariate analysis between ER, PgR or HER2 and survival, although there was a trend towards better survival rates in women with ER+ tumours. A statistically significant positive correlation of cytosolic BAG-1 immunostaining with Bcl-2 expression was found in 62 of 76 (82%) breast tumours coexpressing these proteins, suggesting that BAG-1 and Bcl-2 may be coregulated to some extent in early-stage invasive breast cancers (Turner *et al*, 2001).

Tang *et al* (2004) (Paik *et al*, 2004) also reported strong immunoreactivity for BAG-1 in the cytoplasm but low in the nucleus in high-grade tumours. The authors reasoned that weak nuclear BAG-1 expression observed in this cohort may be due to the presence of a high proportion (58%) of poorly differentiated tumours. No correlation was found between cytoplasmic BAG-1 expression with disease-free or overall survival, and further subgroup analysis was precluded as the power to detect any real difference was deemed quite low. Spearman’s *ρ* analysis revealed a correlation between BAG-1 expression and that of Bcl-2, p53, ER and PgR, and the better differentiation of breast carcinoma. Correlation was significant between BAG-1 expression and that of ER, Bcl-2 pattern and intensity and differentiation in univariate analysis, whereas expression of BAG-1 significantly correlated only with that of ER in multivariate analysis. In contrast, previously published data from the same group (Tang *et al*, 1999) showed no correlation between the expression patterns of BAG-1 and that of ER or PgR in invasive breast carcinoma; this could be due to the lower proportion of ER+ and PgR+ tumours, missing data about receptor status, or the smaller cohort size. However, BAG-1 staining correlated with differentiation. Total BAG-1 staining significantly correlated with shorter disease-free and overall survival in multivariate analysis. Moreover, patients whose tumours had high nuclear BAG-1 expression had a trend towards shorter disease-free and overall survival (Tang *et al*, 1999). These findings are supported by Athanassiadou *et al* (2009), who showed that nuclear expression of BAG-1 was associated with lower survival rates compared with total or cytoplasmic BAG-1 staining. Positive overall staining for BAG-1 was associated with lower 5-year survival rates compared to negative staining. In univariate analysis, nuclear BAG-1 staining was correlated with worse prognostic indicators (stages III–IV, tumour size >5 cm and presence of four or more positive lymph nodes) compared to cytoplasmic staining (stage II, tumour size 2–5 cm and one to four positive lymph nodes). No significant correlation was found between ER and PgR status and BAG-1 staining pattern (Athanassiadou *et al*, 2009).

Nadler *et al* (2008) found no difference in all prognostic variables between nuclear and cytoplasmic BAG-1, but correlated total BAG-1 with significantly improved survival outcomes in node-positive patients by univariate analysis, whereas in multivariate analysis, BAG-1 did not retain its independent prognostic value. Histological grade and treatment information were not given. Moreover, Spearman’s *ρ* analysis revealed a significant association between BAG-1 with Bcl-2, ER and PgR prognostic markers.

Millar *et al* (2009) also found that high levels of nuclear and cytoplasmic BAG-1 were significantly associated with improved prognosis for local recurrence, distant metastases and cancer-specific death in univariate analysis. Nuclear and cytoplasmic BAG-1 expression was associated with low-grade tumours, ER and PgR positivity, and improved overall survival but was negatively correlated with HER2 and the triple-negative phenotype. Subtype analysis revealed that high nuclear BAG-1 expression alone is an independent predictor of outcome of ER+ tumours and correlates strongly with a luminal A intrinsic phenotype in both univariate and multivariate analyses; nuclear BAG-1 staining did not associate with outcome in univariate analysis of ER-negative tumours. Treatment of patients with tumours exhibiting high nuclear BAG-1 expression with tamoxifen showed an improved outcome for local recurrence, distant metastases and breast cancer-specific death (Millar *et al*, 2009). Similar findings were reported by Cutress *et al* (2003), who showed that high nuclear BAG-1 staining is a marker of good prognosis in a relatively homogeneous cohort of node-negative, ER+ patients treated with hormonal therapy (tamoxifen or anastrozole) but not chemotherapy after tumour resection. A strong inverse correlation was found between nuclear BAG-1 expression and tumour size, whereas ER*α* and PgR expression moderately correlated with nuclear and (to a lesser extent) with cytplasmic BAG-1 expression. Taken together, the data by Cutress *et al* (2003) and Millar *et al* (2009) are consistent with the role of BAG-1 as a prognostic biomarker in the oncotype DX assay, and demonstrate that a high nuclear BAG-1 expression identifies a group of breast cancers with good prognosis and with enhanced sensitivity to hormonal therapy.

In line with these data, recent analysis of the TransATAC clinical trial cohort (Afentakis *et al*, 2013) and retrospective (Gee *et al*, 2010) studies in ER+ early breast cancer treated with hormonal therapy but not chemotherapy, show that expression of BAG-1 significantly associates with that of ER and PgR, and correlates with tumour grade. Bcl-2-associated athanogene-1 status is a more powerful marker than either Ki-67 or HER2 in relation to disease-free interval and than HER2 for survival in multivariate analysis (Gee *et al*, 2010). Moreover, nuclear BAG-1 immunoreactivity exhibits significant value for estimating a residual risk that is independent of standard clinical and immunohistochemical parameters, particularly in node-positive patients (Afentakis *et al*, 2013).

### Meta-analyses of BAG-1 expression and outcome

**Figure 2:**
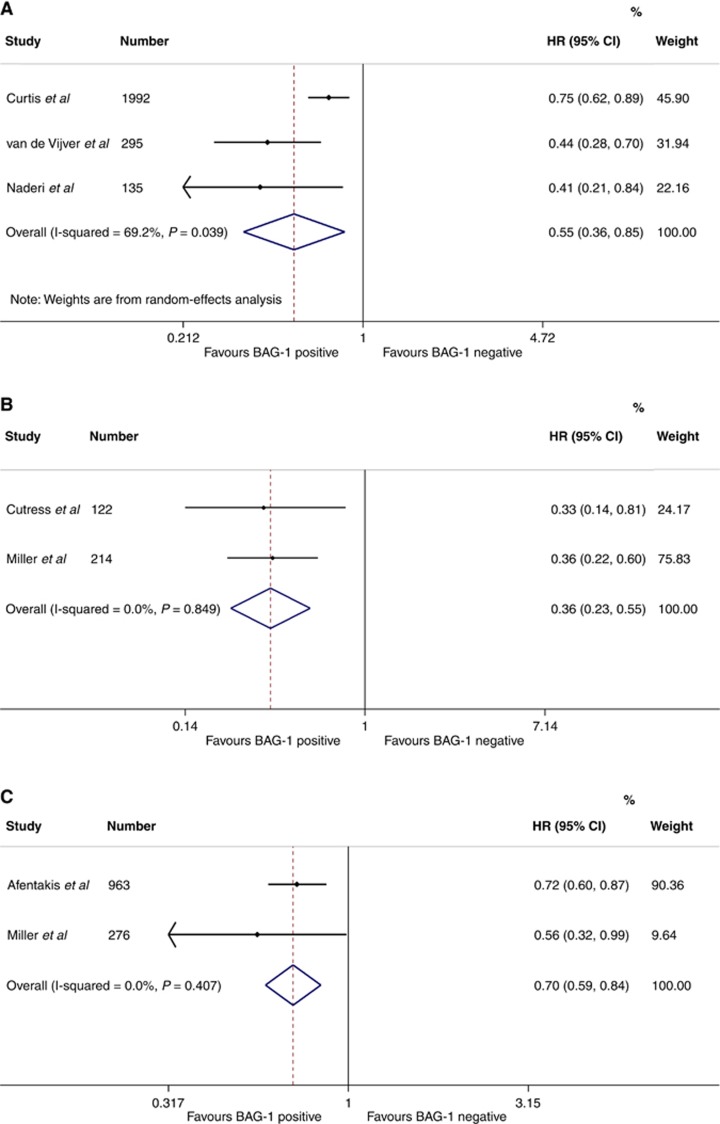
Meta-analyses of: (**A**) BAG-1 mRNA (high v low) for BCSS. Data for van de Vijver *et al.*, and Nadieri *et al.*, are from analyses published in Millar *et al* (2009), and for Curtis *et al.*, are from analyisis included in Papadakis *et al.*, and 95% CI obtained from the authors of Papadakis *et al* (2016) (**B**) nuclear BAG-1 protein by immunohistochemistry (high v low) for BCSS; and (**C**) nuclear BAG-1 protein by immunohistochemistry (high v low) for DDFS.

In general, data were too heterogenous, and outcome measures were too varied to perform meta-analyses for the majority of studies. Meta-analyses of mRNA expression from the two data sets analysed in Millar *et al* (2009) and the data set analysed in Papadakis *et al* (2016) including a total of 2422 patients produced a HR of 0.55 (95% CI 0.36–0.85) favouring improved BCSS with high expression of BAG-1 (Figure 2a). Similarly of the two studies (336 patients; Figure 2b) reporting pathologist assessment of nuclear BAG-1, improved BCSS was observed with high BAG-1 (HR 0.36; 95% CI 0.23–0.55). Nadler *et al* (2008) was not included in this analysis, as a different (automated) method of assessment of BAG-1 expression was used to the other immunohistochemical studies. Sensitivity analysis suggests that the result for nuclear BAG-1 and BCSS becomes non-significant with the inclusion of this study. Of the two studies (1239 patients; Figure 2c) reporting nuclear BAG-1 and DDFS, improved outcome was seen with high BAG-1 (HR 0.70; 95% CI 0.59–0.84).

## Discussion

As previous review (Cutress *et al*, 2002), evidence supporting the hypothesis that BAG-1 plays an important role in breast cancer has increased. The development of high-throughput assays, such as oncotype DX and PAM50, reveal that increased BAG-1 mRNA is associated with a low risk of recurrence and improved prognosis. In addition, a large RT-PCR study of a clinical trial cohort is also consistent with this (Dowsett *et al*, 2015). Similarly, recent retrospective and clinical trial immunohistochemical studies of large patient cohorts show that increased BAG-1 expression associates significantly with that of ER and PgR and with histological grade. Moreover, high nuclear BAG-1 immunoreactivity is an independent predictor of outcome particularly in patients with ER+ early breast cancer receiving adjuvant hormonal therapy, and enhances the predictive power of IH4 staining. Including BAG-1 immunohistochemical staining as a standard biomarker in the clinic may therefore help to better stratify patients according to their risk of disease recurrence and determine their probability of responding to therapy.

A recent study assessed the possibility of performing immunohistochemical staining on a panel of 10 gene products included in Oncotype DX to reduce the number of patients requiring testing due to the increased cost of using this assay (Ingoldsby *et al*, 2013). Classification and regression tree analysis correctly classified 77% of cases into TAILORx categories based on nuclear pleomorphism, survivin, cyclin B1 and BAG-1. Staining ER+ breast cancer subtypes in a clinical pathology laboratory for BAG-1 may therefore help to identify individuals who will respond better to hormonal therapy without the need for unnecessary chemotherapy.

The concept that BAG-1, a protein that supports cancer cell survival, is related to improved patient survival may seem paradoxical. This observation, however, is not without precedent as both the ER, and Bcl-2, another anti-apoptotic protein, is also associated with good prognosis in breast cancer.

The controversy surrounding expression patterns and intensity of BAG-1 staining is apparent. Nevertheless, differences introduced by patient cohort heterogeneity in terms of histological type and number, tumour grade and treatment, the different antigen retrieval methods and antibodies used and the threshold chosen for judging positive staining may account for some of the differences between studies. For example, of studies that utilised an anti-BAG-1 monoclonal antibody, the majority demonstrated either a positive correlation between BAG-1 expression and outcome (Turner *et al*, 2001; Cutress *et al*, 2003; Lin *et al*, 2008; Millar *et al*, 2009; Afentakis *et al*, 2013) or a trend to this that was not significant(Sirvent *et al*, 2004), or a positive correlation in node-positive patients (Nadler *et al*, 2008). In contrast, three immunohistochemical monoclonal antibody studies did not demonstrate a correlation (Tang *et al*, 2004; Yang *et al*, 2008; Wang *et al*, 2014).

Although the meta-analyses, consistent with inclusion of BAG-1 in Oncotype DX and PAM50, suggested association between BAG-1 and clinical outcome, it was not possible to include many of the reported studies in the meta-analyses due to the lack of available data. However, studies that could not be included in the meta-analyses included four that reported significant positive correlations between BAG-1 expression and outcome (Turner *et al*, 2001; Yun *et al*, 2005; Lin *et al*, 2008; Afentakis *et al*, 2013) and two with a trend towards a positive correlation that was not significant (Townsend *et al*, 2002; Sirvent *et al*, 2004), consistent with the meta-analyses. In contrast, five reported no correlation with BAG-1 expression and outcome (O’Driscoll *et al*, 2003; Tang *et al*, 2004; Yang *et al*, 2008; Wang *et al*, 2014; Davidson *et al*, 2016), and two reported a negative correlation with outcome(Tang *et al*, 1999; Athanassiadou *et al*, 2009), one of which was an immunocytochemical rather than immunohistochemical study (Athanassiadou *et al*, 2009). Overall, from all studies, and consistent with the meta-analyses and RT-PCR studies, our interpretation is that the most consistent finding appeared to be a positive correlation with outcome in those with high BAG-1 levels.

To explain the clinical association observed between BAG-1 expression and localisation in breast cancer with other clinicopathological parameters such as ER expression, sensitivity to tamoxifen and prolonged patient survival, some studies have used breast cancer cell line models. The impact of BAG-1 on patient survival may depend partly on the regulation of ER function, particularly at an early stage of the disease. Targeting BAG-1S or BAG-1M to the nucleus fails to enhance ER transcriptional activity; however, BAG-1L is capable of achieving this particularly in the presence of oestrogens. As lifetime exposure to oestrogens is a significant risk factor for breast cancer development, BAG-1L may increase this through its sensitising effects on ER*α* and ER*β*. This notion is supported by evidence that anti-oestrogen therapies alter the sensitivity of BAG-1 overexpressing ER+cells to cell cycle arrest, whereas downregulation of BAG-1 expression enhances the sensitivity of tamoxifen-resistant MCF-7 cells to tamoxifen (Liu *et al*, 2014). It should be noted that all BAG-1 isoforms are produced from a single mRNA, and all antibodies used in these studies recognise all BAG-1 isoforms so it is not possible to comment on the significance of the individual BAG-1 isoforms. It is tempting to speculate based on the cell line evidence that the nuclear localised BAG-1L isoform could be a more powerful progostic and predictive biomarker than total or nuclear BAG-1 expression. Studies utilising BAG-1L-specific antibodies in large patient cohorts stratified based on disease subtype, treatment and clinicopathological characteristics should address this hypothesis.

Although the findings should be interpreted with caution due to the number of studies that could not be included in the meta-analyses, overall and despite heterogeneity between studies, this systematic review and meta-analyses suggest that increased expression of BAG-1 mRNA and BAG-1 protein, and in particular nuclear expression, appears associated with improved breast cancer outcomes.

## Figures and Tables

**Table 1:** Studies of BAG-1 expression in breast cancer

	Tang *et al*, 1999	Turner *et al*, 2001	Townsend *et al*, 2002	Cutress *et al*, 2003	O’Driscoll *et al*, 2003	Tang *et al*, 2004	Sirvent *et al*, 2004	Yun *et al*, 2005	Lin *et al*, 2008	Nadler *et al*, 2008	Yang *et al*, 2008	Millar *et al*, 2009	Athanassiadou *et al*, 2009	Afentakis *et al*, 2013	Wang *et al*, 2014	Dowsett *et al*, 2015	Papadakis *et al*, 2016	Davidson *et al*, 2016
**Introduction**																		
Marker	BAG-1, PR, ER	BAG-1, BCL2, P53, ER, PR	BAG-1, HSC70	BAG-1	BAG-1, survivin, survivin-DEx3, survivin-2B, galectin-3, Bcl-2, MRP-1, Bax-a	BAG-1, Bcl-2, P53	BAG-1, Bcl-2, P53, BAX	BAG-1	BAG-1	BAG-1, Bcl-2	BAG-1	BAG-1	BAG-1, CD24	BAG-1	Bag-1, Parp-1, EGFR	GRB7, HER2, Cyclin B1, Ki-67, MYLB2, STK15, Survivin, BCL2, CUBE2, ER, PgR, Cathepsin L2, Stromelysin, BAG-1, CD68, GSTM1, ACTB, GAPDH, GUS, RPLPO, TFRC	BAG-1	BAG-1, HSP70, HSP90
Objectives	✓	✓	✓	✓	✓	✓	✓	✓	✓	✓	✓	✓	✓	✓	✓	✓	✓	✓
Hypotheses	✓	✓	✓	✓	✓	✓	✓	✓	✓	✓	✓	✓	✓	✓	✓	✓	✓	✓
**Materials and methods**																		
**Patients**																		
Disease stage	I–IV	I–II	IV	I–IV	I–III	I–IV	I–II	I–III	I–IV	I–IV	I–III	I–IV	I–IV	I–III	I–III	I–III	I–III	I–III
Disease subtype	Not limited by specific subtype	Not limited by specific subtype	Not limited by specific subtype	Not limited by specific subtype	Not limited by specific subtype	Not limited by specific subtype	Ductal	Not limited by specific subtype	Not limited by specific subtype	Not limited by specific subtype	Not limited by specific subtype	Ductal	Not limited by specific subtype	ER positive	Not limited by specific subtype	ER positive	Not limited by specific subtype	Not limited by specific subtype
Co-morbidities	×	×	×	×	×	×	×	×	×	×	×	×	×	×	×	×	×	×
Inclusion/exclusion criteria	✓	✓	✓	✓	✓	✓	✓	✓	✓	✓	✓	✓	✓	✓	✓	✓	✓	✓
Treatment received	Not limited by specific treatment	Not limited by specific treatment	Not limited by specific treatment	Not limited by specific treatment	Not limited by specific treatment	Not limited by specific treatment	Not limited by specific treatment	Not limited by specific treatment	Not limited by specific treatment	Not limited by specific treatment	Not limited by specific treatment	Not limited by specific treatment	Not limited by specific treatment	Tamoxifen or anastrazole	Not limited by specific treatment	Tamoxifen or anastrazole	Not limited by specific treatment	Not limited by specific treatment
Treatment randomised	×	×	×	×	×	×	×	×	×	×	×	×	×	×	×	×	×	×
**Specimen**																		
Type	PTS	PTS	PTS	PTS	PTS	PTS	PTS	PTS	PTS	PTS	PTS	PTS	PTS	PTS	PTS	PTS	PTS	PTS
Controls	PTN	N	PTN	PTN	N	PTN	PTN	N	PTN	PTN	PTN	N	PTN	PTN	PTN	PTN	N	PTN
**Assay**																		
Tissue sample	Whole sections+IHC	Whole sections+IHC	Whole sections+IHC	Whole sections+IHC	Whole sections+RT-PCR	Whole sections+IHC	Whole sections+IHC	Whole sections+IHC	Microarray	Microarrary	Microarrary	Microarray	Imprint smear+IHC	Microarrary	Microarrary	Whole sections+RT-PCR	Whole sections+RT-PCR	Microarray
Antibody	Santa Cruz, polyclonal, rabbit, (C16): sc-939	Dako, monoclonal, mouse, KS6C8	Santa Cruz, polyclonal, rabbit, C12 & TB2	Dako, monoclonal, mouse, KS6C8	RT-PCR	Santa Cruz, polyclonal, rabbit, (C16)sc-939	Neomarkers, monoclonal, mouse, 3.10G3E2	Wuhan Boster, polyclonal, rabbit	Wuhan Boster, polyclonal, rabbit	Dako, monoclonal, mouse, KS6C8	Zhongshang, monoclonal, mouse	Santa Cruz, monoclonal, mouse, 3.10G3E2	Novocastra, monoclonal, mouse, 5C5	Dako, monoclonal, mouse, 3.10G3E2	Abcam, polyclonal, rabbit, ab112493	RT-PCR	RT-PCR	Santa Cruz, polyclonal, rabbit
Histopathology score	H-score	H-score	H-score	H-score	RT-PCR	H-score	H-score	H-score	H-score	AQUA score	H-score	H-score	H-score	H-score	H-score	RT-PCR	RT-PCR	H-score
Controls	✓	✓	✓	✓	✓	✓	✓	✓	✓	✓	✓	✓	✓	✓	✓	✓	✓	✓
Blinded?	×	✓	×	×	×	✓	×	✓	✓	✓	✓	✓	✓	✓	✓	✓	×	✓
**Study**																		
Type	R	R	R	R	R	R	R	R	R	R	R	R	R	P	R	R	R	R
Period of follow-up in years (median)	8	12.1	12.8	5.6	6	3.7	4.5	×	×	10	5	5.3	3.3	10	9.1	10	×	19
Clinical end points defined	DFS, OS	DDFS, OS, BCSS	DFS, OS	BCSS, OS	DFS, OS	DFS, OS	DFS, OS	OS	OS	BCSS	OS	BCSS, LR, DDFS	OS	DFS, DDFS, OS	OS	DDFS, LR, NPC, OS	OS	OS
Rationale for sample size	✓	✓	✓	✓	✓	✓	✓	✓	✓	✓	✓	✓	✓	✓	✓	✓	✓	✓
Study power, *N*	140	122	160	138	106	185	186	100	100	638	78	292	70	963	119	1125	1971	410
**Statistical analysis**																		
Method	Cox-hazard, Kaplan–Meier	Multivariate Cox proportional hazards models	*χ*^2^, Kaplan–Meier, log-rank	Cox-hazard, Kaplan–Meier	Pearson’s *χ*^2^, Kaplein–Meier	Spearman *ρ*, logistic regression	Cox-hazard and Kaplan–Meier	Fisher’s exact test	Pearson’s *χ*^2^	Cox univariate and multivariate	Pearson’s *χ*^2^, Cox-hazard	*χ*^2^, Cox-hazard and Kaplan–Meier	*χ*^2^, Cox-hazard and Kaplan–Meier	Spearmans, *χ*^2^ likelhood ratio, Kaplein–Meier	Cox-hazard and Kaplan–Meier	Cox-hazard, *χ*^2^ likelhood ratio	Kaplan–Meier and Cox regression	Kaplan–Meier and Cox regression
**Results**																		
**Data**																		
Flowchart of patient flow through study	×	×	×	×	×	×	×	×	×	×	×	×	×	✓	×	✓	×	×
Prognostic variables	✓	✓	✓	✓	✓	✓	✓	✓	✓	✓	✓	✓	✓	✓	✓	✓	✓	✓
Demographic characteristics	✓	✓	✓	✓	✓	✓	✓	✓	✓	✓	✓	✓	✓	✓	×	✓	✓	✓
Missing data	✓	✓	✓	×	✓	✓	✓	✓	✓	✓	×	✓	×	×	✓		✓	✓
**Analysis**																		
Relation of marker to prognostic variables	✓	✓	✓	✓	✓	✓	✓	✓	✓	✓	✓	✓	✓	✓	✓	✓	✓	✓
Univariate analysis between marker and outcome (*P* value, hazard ratio, confidence interval)	✓	✓	✓	✓	✓	✓	✓	✓	✓	✓	✓	✓	✓	✓	✓	✓	✓	✓
Multivariate analyses (*P* value, hazard ratio, confidence interval)	✓	✓	✓	✓	✓	✓	✓	✓	✓	✓	✓	✓	✓	✓	✓	✓	✓	×
State confidence intervals	×	×	×	✓	×	×	✓	×	×	✓	✓	✓	×	✓	✓	✓	×	×
Report any results of further investigations	×	×	×	×	×	×	×	×	×	×	✓	✓	✓	×	✓	✓	×	✓
**Discussion**																		
Interpret in context of hypotheses	✓	✓	✓	✓	✓	✓	✓	✓	✓	✓	✓	✓	✓	✓	✓	✓	✓	✓
Implications for future research and clinical value	✓	✓	✓	✓	✓	✓	✓	✓	✓	✓	✓	✓	✓	✓	✓	✓	✓	✓

Abbreviations: BCSS=breast cancer-specific survival; DDFS=distant disease-free survival; DFS=disease-free survival; IHC=immunohistochemistry; LR=local recurrence; NPBC=new primary breast cancer; OS=overall survival; P=prospective; PTN=peritumoural normal tissue; PTS=Primary tumour specimen; R=retrospective.

Study compliance with REMARK criteria.

**Table 2a:** Immunohistochemical studies showing level of BAG-1 expression in breast cancer and relationship with prognostic markers

		**Total BAG-1 staining (%)**			**Subcellular BAG-1 staining (%)**			**Relationship with prognostic markers**		
**Reference**	**BAG-1-positive samples (%)**	**Weak**	**Moderate**	**Strong**	**Nuclear**	**Cytoplasmic**	**Mixed**	**Correlation**	**Univariate** ***P*** **(N/C/B)**	**Multivariate** ***P*** **(N/C/B)**
Tang *et al*, 1999	77.1	23.6	35.7	17.9	18.2	57.1	1.4	Negative	NS	B: *P*=0.0052 DFS; *P*=0.0033 OS
Turner *et al*, 2001	NG	NG	NG	NG	23.0	65.0	NG	Positive	C: *P*<0.001 DDFS, BCSS & OS	C: *P*=0.005 DDFS, *P*=0.008 BCSS, *P*=0.01 OS
Townsend *et al*, 2002	92.0	NG	NG	NG	47.0	84.0	NG	Trend to positive	NS	NS
Cutress *et al*, 2003	NG	NG	NG	NG	54.0	22.1	NG	Positive	N: *P*=0.015 BCSS	NS
Tang *et al*, 2004	86.0	61.0	NG	25.0	0.5	85.5	NG	NS	NS	NS
Sirvent *et al*, 2004	80.6	NG	NG	NG	NG	NG	NG	Trend to positive	NS	NS
Yun *et al*, 2005	85.0	NG	NG	NG	NG	NG	NG	Positive	*P*=0.04 OS	NS
Lin *et al*, 2008	NG	NG	NG	NG	NG	NG	NG	Positive	*P*<0.01 DDFS	NS
Nadler *et al*, 2008	NG	NG	22.0	78.0	NG	NG	NG	Overall NS, positive in node positive	NS	NS
Yang *et al* 2008	76.0	34.0	29.0	13.0	NG	NG	NG	NS	NS	NS
Millar *et al*, 2009	NG	NG	NG	NG	54.0	63.0	NG	Postive	N: *P*=0.002 LR, *P*<0.0001 DDFS & BCSS	N: *P*=0.0455 DDFS
Athanassiadou *et al*, 2009	70.0	NG	NG	NG	27.1	51.4	8.6	Negative	B & N: *P*<0.0001, C 0.002 OS	NS
Afentakis *et al*, 2013	NG	NG	NG	NG	NG	NG	NG	Positive	N: *P*=0.0005, C: *P*=0.0007 DFS; N: *P*=0.0006, C *P*=0.001 DDFS	BAG-1 N added to IHC4
Wang *et al*, 2014	95.8	NG	NG	NG	NG	NG	NG	NS	NS	NS
Davidson *et al*, 2016	48.0	29.8	9.5	1.4	NG	NG	NG	NS	NS	NS

Abbreviations: BCSS=breast cancer-specific survival; DDFS=distant disease-free survival; DFS=disease-free survival; LR=local recurrence; N/C/B=nuclear/cytoplasmic/overall; NG=not given; NS=not significant; OS=overall survival.

In this table, a positive correlation indicates that higher levels of BAG-1 expression are associated with improved breast cancer outcome and a negative correlation with poorer breast cancer outcome. Where a there is a statistically significant finding further details are provided.

**Table 2b:** RT-PCR studies showing relationship between BAG-1 expression and prognostic markers

		**Relationship with prognostic markers**		
**Reference**	**BAG-1-positive samples (%)**	**Correlation**	**Univariate** ***P*** **(N/C/B)**	**Multivariate** ***P*** **(N/C/B)**
O’Driscoll *et al*, 2003	80.9	NS	NS	NS
Millar *et al*, 2009 (using data from van de Vijver *et al*, 2002)	79.3	Positive	OS; *P*=0.005	NS
Millar *et al*, 2009 (using data from Naderi *et al*, 2007)	80.0	Positive	OS; *P*=0.0120/0.0151	NS
Dowsett *et al*, 2015	NG	Positive	AR; HR: 0.70; 95% CI: 0.58–0.85 DR; HR: 0.66; 95% CI: 0.53–0.83	NG
Papadakis *et al*, 2016 (using data from Curtis *et al*, 2012)	NG	Positive	BCSS; *P*=0.001	BCSS; *P*=0.022 HR: 0.81; 95% CI: 0.67–0.97

Abbreviations: AR=any recurrence; BCSS=breast cancer-specific survival; CI=confidence interval; DR=distant recurrence; HR=hazard ratio; NG=not given; NS=not significant; OS=overall survival.
